# QiDiTangShen Granules Reduced Diabetic Kidney Injury by Regulating the Phosphorylation Balance of the Tyrosine and Serine Residues of Insulin Receptor Substrate 1

**DOI:** 10.1155/2018/2503849

**Published:** 2018-06-28

**Authors:** Xue Gao, Hongfang Liu, Zhichao An, Qiying He

**Affiliations:** Dongzhimen Hospital, Beijing University of Traditional Chinese Medicine, Beijing 100700, China

## Abstract

**Background:**

Diabetic nephropathy (DN) is a microvascular complication induced by diabetes mellitus (DM), which can affect life quality and long-term prognosis of patients with DM. Angiotensin-converting-enzyme inhibitors (ACEI)/angiotensin receptor blockers (ARB) are currently recommended for treating DN proteinuria, but patients receiving ACEI/ARB are at risk of elevated serum creatinine or potassium levels. Based on the “yin-yang” theory of traditional Chinese medicine, the present study explored the effect of QiDiTangShen (QDTS) granules on DN and the phosphorylation balance of tyrosine and serine residues of IRS-1.

**Methods:**

In this experiment, db/db mice were used as an animal model for type 2 diabetic nephropathy. The intervention (QDTS granules and valsartan) started when the mice were 12 weeks old. C57BL/6 mice were used as normal control. The urine albumin excretion ratio (UAER) was measured by enzyme-linked immunosorbent assay (ELISA) before and after the intervention. The IRS-1, PI3K, Akt, and MAPK proteins expression and the phosphorylation levels were detected by western blot.

**Results:**

QDTS granules reduced the 24-h urinary albumin excretion rate (UAE) in db/db mice with type 2 DM and attenuated the pathological changes of the kidney. QDTS granules also increased the activation level of the PI3K/Akt signaling pathway and reduced insulin resistance. In addition, QDTS granules inhibited the activation of ERK and p38MAPK and decreased the phosphorylation ratio of Ser307/Tyr896 of IRS-1 in renal tissue.

**Conclusions:**

QDTS granules reduced DM-induced renal injury by improving insulin sensitivity via suppressing MAPK signaling and restoring the phosphorylation balance of tyrosine/serine of IRS-1.

## 1. Introduction

Diabetic nephropathy (DN) is a microvascular complication induced by diabetes mellitus (DM). It is the leading cause of end-stage renal disease (ESRD) and death in patients with DM [1]. Multiple metabolic and inflammatory factors are involved in the functional impairment of the renal microvascular barrier [2, 3], which subsequently causes the formation of DN albuminuria [4].

Angiotensin-converting-enzyme inhibitors (ACEI)/angiotensin receptor blockers (ARB) are the only drug classes recommended by ADA for clinical use to control DN urinary albumin excretion and disease progression [5]. However, it remains a challenge that ACEI/ARB could potentially increase the risk of elevated serum creatinine level or hyperkalemia. Further, ADA does not recommend ACEI/ARB as a prophylactic treatment in patients without microalbuminuria. In addition, there is no clinical evidence that ACEI/ARB can improve renal prognosis in patients with DM and without hypertension. Currently, there is no recognized alternative therapy for treating DN in patients without the indications for ACEI/ARB.

Abundant evidence has confirmed that insulin resistance is associated with the development and progression of DN [6]. Renal podocyte specific insulin receptor gene knockout mice can present typical DN pathological changes, while the systemic blood glucose remains normal [7]. PI3K/Akt is one of the main downstream pathways for intracellular transduction of insulin signaling, which can be activated by the tyrosine phosphorylated insulin receptor substrate 1 (IRS-1) [8, 9]. The MAPK signaling pathway can inhibit the phosphorylation of tyrosine residues of IRS-1 by promoting the phosphorylation of the serine residues, thereby hindering the signal transduction of insulin [10, 11]. Therefore, by regulating the activation of the PI3K/Akt and MAPK signaling pathways and restoring the phosphorylation balance of the tyrosine and serine residues of IRS-1 may attenuate diabetic kidney injury.

In traditional Chinese medicine (TCM), “yin” and “yang” represent two different types of functions. “Yin” stands for inhibition and inactivation, and “yang” stands for promotion and stimulation; “yin” and “yang” can also be described as negative and positive feedback or regulatory networks [12]. The essential way of treating disease in TCM is to adjust the balance between “yin” and “yang”, including the balance between inhibition and promotion and inactivation and stimulation. QiDiTangShen (QDTS) granules, containing Dihuang (*Rehmannia glutinosa*), are used to “supplement the kidney essence” and “dredge the collaterals”. Our previous clinical trials confirmed that QDTS granules could effectively reduce proteinuria in patients with DN [13]. It has been reported that a TCM compound preparation with* R. glutinosa* as the main medicinal ingredient is able to promote the activation of PI3K/Akt signaling pathway of type 2 diabetic rats and reduce insulin resistance [14].

The db/db mouse is an animal model for spontaneous type 2 DM. These animals are obese, hyperinsulinemic, and hyperglycemic [15]. Db/db mice are widely used to study the pathogenesis and treatment of DN. Many studies have found that the content of various miRNA in renal tissues of db/db mice has changed and is related to the occurrence of kidney injury. The elevated miR-134-5p could promote podocyte apoptosis by targeting BCL2 [16], and the downregulation of miR-30c was closely related to epithelial-to-mesenchymal transition (EMT) and renal fibrosis [17]. Zhang et al. found 355 differentially expressed genes (DEGs) by microarray analysis, which are mainly distributed in the three major significant pathways of biological oxidation, bile acid metabolism, and steroid hormone synthesis [18]. In addition, various interventions can reduce renal damage in db/db mice by regulating podocyte autophagy activities [19] and antifibrosis effect [20]. Thus, we chose db/db mice to explore the effect of QDTS granules on DN and on the phosphorylation levels of tyrosine/serine residues of IRS-1.

## 2. Materials and Methods

### 2.1. Animal Model and Drug Administration

Twenty-four male db/db mice (8 weeks old) and eight C57BL/6 mice (8 weeks old) were purchased from Beijing Vital River Laboratory Animal Technology Co., Ltd. (certificate number: SCXK [Beijing] 2012-0001). This study was carried out in the animal laboratory with barrier facilities (certificate number: SYXK [Beijing] 2015-0001) of Dongzhimen Hospital Affiliated to Beijing University of Chinese Medicine. All animals were handled according to the guidelines of the Beijing University of Chinese Medicine Animal Research Committee. The experimental protocol was approved by the Laboratory Animal Welfare and Ethics Committee of Beijing University of Chinese Medicine (certificate number: 16-14). QDTS granules are composed of Dihuang (*R. glutinosa*), Huangqi (Mongolian milkvetch,* Astragalus propinquus*), Qianshi (seeds of fox nut,* Euryale ferox*), Shanzhuyu (Japanese cornel,* Cornus officinalis*), Shuizhi (leeches,* Whitmania pigra*), Dahuang (Chinese rhubarb,* Rheum officinale*), and Baihuasheshecao (*Hedyotis diffusa*). The water extract of this prescription was provided by Zhuozhoudongle Pharmaceutical Co., Ltd. (Hebei, China). The adult dosage of the water extract of QDTS granules was 26.2 g/day. Valsartan capsules (Diovan®, 80 mg × 7 tablets, Beijing Novartis Pharmaceutical Co., Ltd.) were used as the control, and the adult dosage was 80 mg/day.

After 4 weeks of adaptive feeding, all mice were divided into four groups according to blood glucose, body weight, and 24-h urinary albumin excretion rate (UAE): C57BL/6 group (as the normal control), db/db group (as the DN model group), db/db + valsartan group and db/db + QDTS group, 8 mice in each group. The mice were housed in cages (temperature 22-24°C, humidity 35-45%, under a 12-h light/dark cycle) and given standard diet and water* ad libitum*. The db/db + valsartan group was given valsartan 10.29 mg/kg/day, and the db/db + QDTS group was given the water extract of QDTS granules 3.37 g/kg/day; each dosage was the equivalent of the human adult dosage. The C57BL/6 group and the db/db group were given the same volume of the vehicle. All treatments were administered once a day (in the morning) by gavage for a continuous 12 weeks.

### 2.2. Body Weight, Blood Glucose, and Insulin Resistance Index (HOMA-IR)

Body weight and random blood glucose were measured every two weeks. Blood glucose was measured by OneTouch® Ultra® Glucometer (Johnson & Johnson, USA) using the blood collected by cutting the tip of the tail. Fasting blood glucose and fasting serum insulin were measured by glucose oxidase and radioimmunoassay (XH-6020 automatic radioimmunoassay counter, Xi'an Nuclear Instrument Factory, China) separately at the end of the experiment. Homeostasis model assessment-insulin resistance (HOMA-IR) was calculated according to the following formula: fasting plasma glucose (FPG, mmol/L) × fasting insulin (FINS, mIU/L)/22.5.

### 2.3. 24-h Urinary Albumin Excretion Rate (UAE)

UAE was measured every four weeks from the starting of the experiment. All of the mice were transferred into the metabolic cage (no feed or water was provided) and 24-h urine was collected. The total amount of the urine was measured, and after centrifugation (3000 rpm, 10 min), the supernatant was stored at −80°C. Urinary albumin was detected by using mice albumin ELISA (enzyme-linked immunosorbent assay) Kit (R&D systems, Minnesota, USA). UAE was defined as urinary albuminuria (*µ*g/mL) × urine volume (mL).

### 2.4. Serum Creatinine (Cr), Urea Nitrogen (BUN), and Uric Acid (UA)

Cr, BUN, and UA were measured to assess the renal function. At the end of the experiment, blood samples were obtained by enucleating the eyeballs. Serum was isolated by centrifugation (3800 rpm, 10 min). The serum Cr, BUN, and UA were analyzed by automatic biochemical analyzer (Olympus AU480, Japan).

### 2.5. Pathological Examination of the Kidney

At the end of the experiment, the left kidney was removed, fixed in 4% paraformaldehyde (PFA), and embedded in paraffin. Sections about 2-3 *μ*m thick were cut and mounted on glass slides, and the paraffin was removed by xylene. The sections were then subjected to Hematoxylin and Eosin (HE) staining and Masson's trichrome staining. The sections were observed using light microscopy with the investigators blinded to the group identity. The right kidney was kept in liquid nitrogen for later use.

Calculation of the percentage of renal fibrosis was conducted as described here: in each section, 10 randomly selected fields were observed under 200x magnification. The ratio of the blue areas of the collagen fibers to the total area was determined as a percentage in each field by Image Pro Plus 6.0 and the mean was calculated [21].

### 2.6. Western Blotting Assay

Proteins extracted from the kidney tissue (stored in liquid nitrogen) were separated on 10% SDS-polyacrylamide electrophoresis gels and then transferred to polyvinylidene fluoride (PVDF) membranes. After being blocked with 5% nonfat milk in TBST for 2 h, the PVDF membrane was incubated with the following primary antibodies overnight: IRS-1, IRS-1 (Ser307), Akt, p-Akt, ERK, and p-ERK antibody (CST, Massachusetts, USA); IRS-1 (Tyr896), PI3K, p-PI3K, p38MAPK, p-p38MAPK, JNK, and p-JNK antibody (abcam, Cambridge, UK); IRS-1 (Ser302) antibody (SAB, Maryland, USA). Next the membranes were washed with 1x TBST for three times, each time for 8 min and then incubated with biotin-labeled goat anti-rabbit or goat anti-mouse IgG (1:5000) at room temperature for 1 h. 5% milk was only used for nonphosphorylated primary antibodies; 3% BSA was used for membrane blocking and for dilution of all phosphorylated antibodies. The ECL hypersensitive luminescent liquid A and B (equal volume) was mixed, and the membrane was incubated with the mixed liquid for 2 min in the dark. The membrane was then placed in a gel imager for signal detection. The image was analyzed with Image Pro Plus 6.0. *β*-actin was used as a loading control for comparison between samples.

### 2.7. Statistical Analysis

All data are presented as mean ± standard error (SEM). Data were analyzed by SPSS22.0 statistical software. One-way analysis of variance was performed to test the differences between the groups. For groups having equal variances, the LSD (least significant difference) method was used; for those having unequal variances, Dunnett's T3 was used.* P* < 0.05 was considered statistically significant.

## 3. Results

### 3.1. QDTS Granules Reduced Insulin Resistance in Type 2 Diabetic db/db Mice

**Figure 1:**
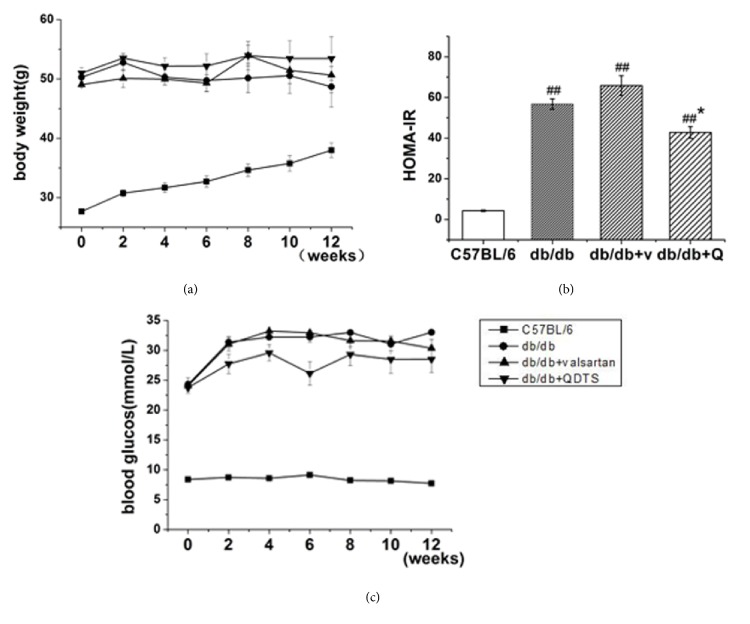
Comparison of body weight, blood glucose, and insulin resistance index of mice in different groups. (a) Body weight. (b) Homeostasis model assessment-insulin resistance (HOMA-IR). (c) Level of blood glucose. HOMA-IR = fasting plasma glucose × fasting insulin/22.5. C57BL/6, the normal control group; db/db, the diabetic nephropathy (DN) model group; db/db + v and db/db + valsartan, the valsartan intervention group; db/db + Q and db/db + QDTS, the QDTS granules intervention group. n = 8 for each group. ^##^*P* < 0.01 compared with the normal control, ^*∗*^*P* < 0.05 compared with the DN model group.

In the present study, the body weight and the random blood glucose were measured every two weeks. The plasma fasting insulin and fasting blood glucose were measured at the end of the twelfth week (i.e., the end of the treatment), and HOMA-IR was calculated. The results showed that compared with the C57BL/6 mice, the body weight and the level of blood glucose of the db/db mice increased, and the HOMA-IR elevated significantly (*P* < 0.01). Compared with the db/db group, the HOMA-IR of the db/db + QDTS group was significantly decreased, which indicated that the insulin resistance was reduced (Figure 1(b)). However, there were no significant changes in body weight and blood glucose level after the intervention of valsartan or QDTS (Figures 1(a) and 1(c)).

### 3.2. QDTS Granules Reduced the UAE of db/db Mice and Decreased Uric Acid

**Figure 2:**
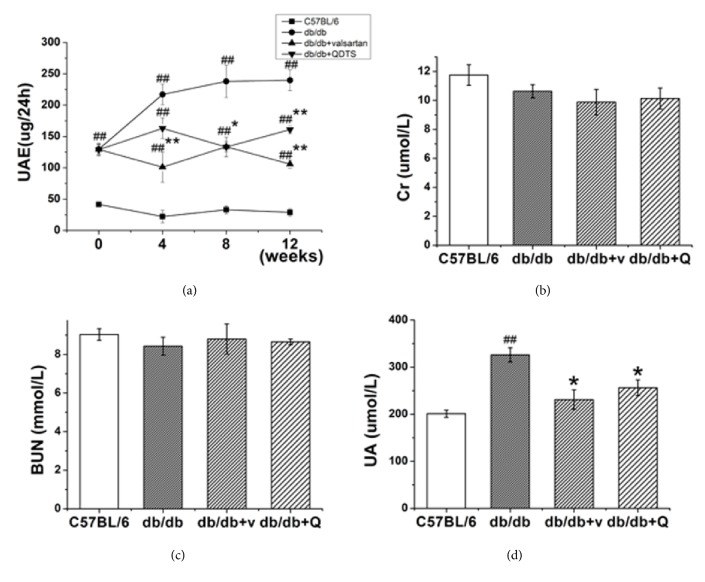
Comparison of 24-h urinary albumin excretion rate (UAE) and renal function of mice in different groups. (a) 24-h UAE. (b) Serum creatinine (Cr) level. (c) Urea nitrogen (BUN) level. (d) Uric acid (UA) level. 24-h UAE was measured every four weeks and Cr, BUN, and UA were measured at the end of treatment. C57BL/6, the normal control group; db/db, the diabetic nephropathy (DN) model group; db/db + v and db/db + valsartan, the valsartan intervention group; db/db + Q and db/db + QDTS, the QDTS granules intervention group. n = 8 for each group. ^##^*P* < 0.01 compared with the normal control, ^*∗*^*P* < 0.05 compared with the DN model group. ^*∗∗*^*P* < 0.01 compared with the DN model group.

Microalbuminuria is the earliest clinical manifestation of DN, which will continuously advance to severe proteinuria and renal failure if not controlled properly. What is more, albuminuria is also an independent risk factor for accelerating the progression of DN [22]. Therefore, reducing UAE is of great importance for DN treatment. We measured UAE every four weeks from the beginning of intervention. The results showed that compared with the C57BL/6 mice, the UAE of the db/db mice was significantly increased. After the intervention, the UAE in the db/db + valsartan group and the db/db + QDTS group was significantly lower than that of the disease model (Figure 2(a)). Although the UAE in the db/db + valsartan group started to decrease earlier than the db/db + QDTS group, there was no significant difference in UAE between the two groups at the end of the treatment, indicating that QDTS granules were similar to valsartan in controlling albuminuria.

At the end of the experiment, we measured the serum Cr, BUN, and UA levels. The results showed that there was no significant difference in serum Cr and BUN between groups (Figures 2(b) and 2(c)). However, the level of UA in the db/db group was significantly higher than that in the C57BL/6 group, and valsartan and QDTS granules could effectively reduce the level of UA (Figure 2(d)).

### 3.3. QDTS Granules Reduced Kidney Pathological Injury of db/db Mice

**Figure 3:**
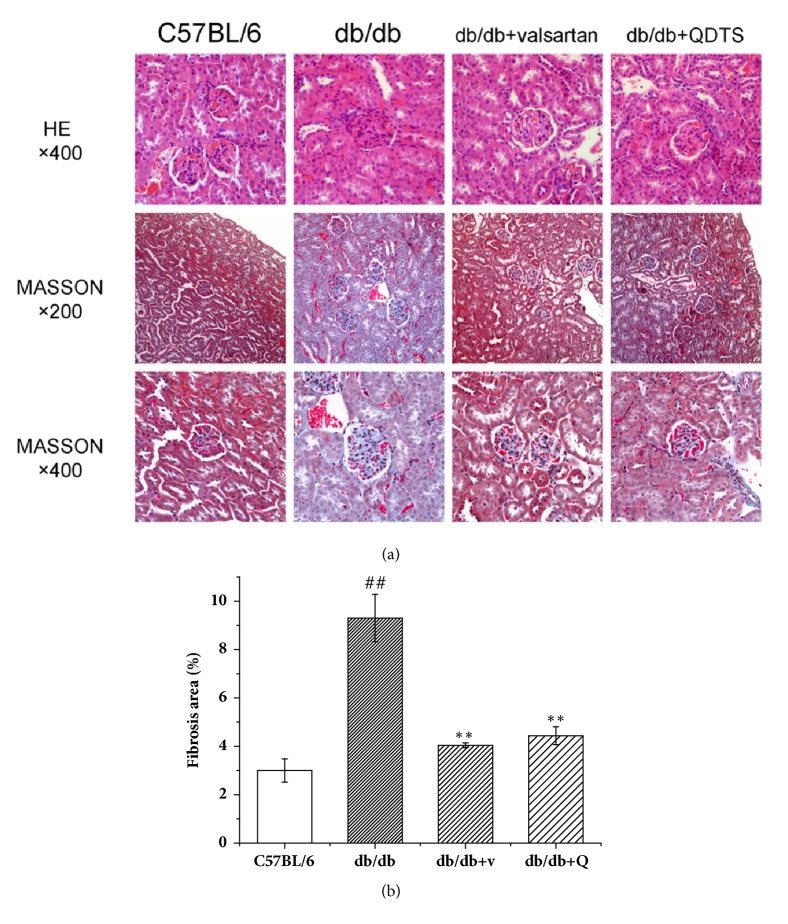
Morphological changes of the kidney of mice in different groups. (a) HE and Masson's trichrome staining of renal sections. (b) Percentage of areas showing fibrosis. Sections stained with Masson's trichrome staining were used to calculate the percentage of areas showing renal fibrosis. At 200x magnification, 10 nonoverlapping fields showing the renal interstitium were selected in each section, the ratio of the blue areas indicating the collagen fibers to the total area was calculated, and the mean value of each treatment group was compared. C57BL/6, the normal control group; db/db, the diabetic nephropathy (DN) model group; db/db + v and db/db + valsartan, the valsartan intervention group; db/db + Q and db/db + QDTS, the QDTS granules intervention group. n = 3 for each group. ^##^*P* < 0.01 compared with the normal control, ^*∗∗*^*P* < 0.01 compared with the DN model group.

According to the HE staining, the morphological structure of the glomeruli and the renal tubules in the renal cortex was normal for mice in the C57BL/6 group. Masson's trichrome staining showed that the blue areas of the collagen fibers mainly located in the renal tubular basement membrane, the mesangial region, and around the peritubular capillaries (Figure 3(a)).

The renal tissue in the db/db group showed glomerular capillary loop hypertrophy and increased mesangial matrix, decreased Bowman's capsule cavity, and vacuolar degeneration of the renal tubular epithelial cells (Figure 3(a)). After the intervention of valsartan and QDTS granules, the pathological changes of the renal cortex were reduced in different degrees (Figure 3(a)). Masson's trichrome staining showed that focal fibrosis was found in the interstitium of the db/db mice, and the percentage of fibrosis in the db/db group was significantly higher than that in the C57BL/6 group (Figure 3(b)). However, the intervention of valsartan or QDTS granules lessened the degree of renal fibrosis of the db/db mice.

### 3.4. QDTS Granules Promoted the Activation of PI3K/Akt Signaling Pathway in the Kidney of the db/db Mice

**Figure 4:**
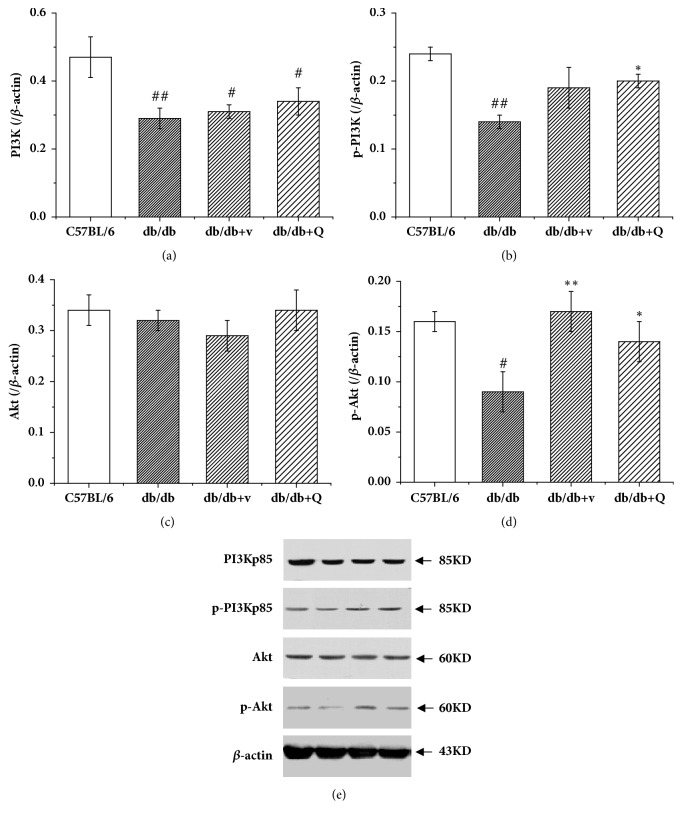
QDTS granules promoted the activation of PI3K/Akt signaling pathway in the kidney of the db/db mice. (a) Expression of PI3K. (b) Expression of phosphorylated PI3K. (c) Expression of Akt. (d) Expression of phosphorylated Akt. (e) Western blot of the expression of PI3K, phosphorylated PI3K, Akt, and phosphorylated Akt. Lane 1, the C57BL/6 group; Lane 2, the db/db model group; Lane 3, the db/db + v group; Lane 4, the db/db + Q group. C57BL/6, the normal control group; db/db, the diabetic nephropathy (DN) model group; db/db + v, the valsartan intervention group; db/db + Q, the QDTS granules intervention group. n = 4 for each group. ^#^*P* < 0.05 compared with the normal control, ^##^*P* < 0.01 compared with the normal control, ^*∗*^*P* < 0.05 compared with the DN model group, and ^*∗∗*^*P* < 0.01 compared with the DN model group.

The expression and phosphorylation levels of PI3K and Akt proteins in renal tissues were detected by western blot. The results showed that the expressions of PI3K as well as the phosphorylated forms of PI3K and Akt in renal tissues of the db/db mice were significantly decreased compared with the C57BL/6 mice (Figures 4(a), 4(b), and 4(d)), indicating that the activation of PI3K/Akt was inhibited under the condition of type 2 DM. The results also showed that QDTS granules increased the phosphorylation levels of PI3K and Akt in the db/db mice, thereby promoting the transduction of insulin signaling (Figures 4(b) and 4(d)), though the treatment did not increase the expression of PI3K and Akt (Figures 4(a) and 4(c)). Valsartan could also improve the phosphorylation of Akt, which was consistent with previous findings [23].

### 3.5. QDTS Granules Restored the Phosphorylation Balance of the Tyrosine and Serine Residues of IRS-1

**Figure 5:**
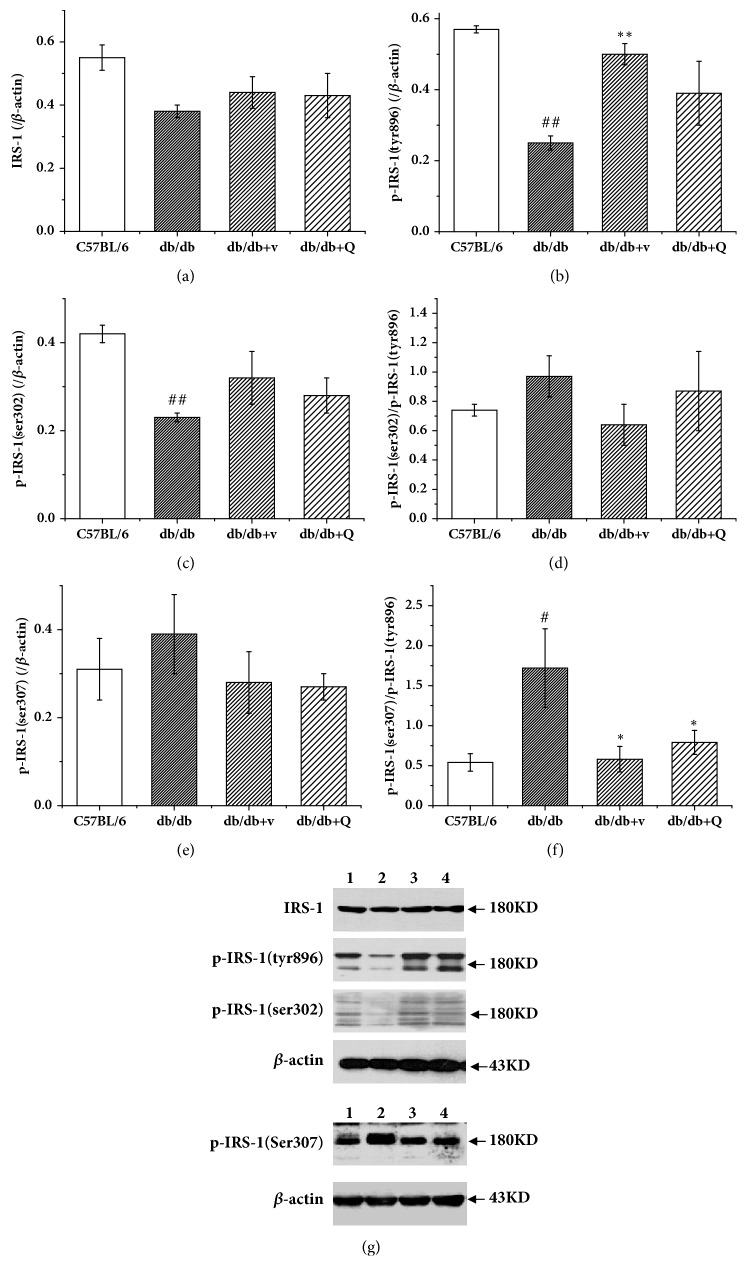
QDTS granules reduced phosphorylation of the serine residues of IRS-1 and restored the phosphorylation balance of tyrosine/serine residues of IRS-1 in kidney tissues of the db/db mice. (a) Expression of IRS-1. (b) Expression of phosphorylated Tyr896 of IRS-1. (c) Expression of phosphorylated Ser302 of IRS-1. (d) Ratio of phosphorylated Ser302/Tyr896 of IRS-1. (e) Expression of phosphorylated Ser307 of IRS-1. (f) Ratio of phosphorylated Ser307/Tyr896 of IRS-1. (g) Western blot of the expression of IRS-1 and the phosphorylated tyrosine and serine residues of IRS-1. Lane 1, the C57BL/6 group; Lane 2, the db/db model group; Lane 3, the db/db + v group; Lane 4, the db/db + Q group. C57BL/6, the normal control group; db/db, the diabetic nephropathy (DN) model group; db/db + v, the valsartan intervention group; db/db + Q, the QDTS granules intervention group. n = 4 for each group. ^#^*P* < 0.05 compared with the normal control, ^##^*P* < 0.01 compared with the normal control, ^*∗*^*P* < 0.05 compared with the DN model group, and ^*∗∗*^*P* < 0.01 compared with the DN model group.

Since the phosphorylation of IRS-1 is the key process in insulin signaling [24], we detected the expression of IRS-1 and the phosphorylation of Tyr896, Ser302, and Ser307 of IRS-1 in renal tissues by western blot. Compared with the C57BL/6 mice, the protein expression of IRS-1 was preserved in the db/db mice (Figure 5(a)). However, the phosphorylation level was significantly decreased for Tyr896 (Figure 5(b)) and Ser302 (Figure 5(c)). Although the phosphorylation level of Ser307 did not change significantly (Figure 5(e)), the ratio of phosphorylated ser307/tyr896 increased significantly (Figure 5(f)), while the ratio of phosphorylated Ser302/Tyr896 showed no obvious change (Figure 5(d)). QDTS granule did not reduce the phosphorylation level of Ser307 (Figure 5(e)), but it reduced the phosphorylation of ser307/tyr896 in db/db mice (Figure 5(f)), while valsartan achieved the same effect by improving the phosphorylation of Tyr896 instead of inhibiting the phosphorylation of Ser307. However, both QDTS granules and valsartan had no significant effect on the phosphorylation of Ser302 of IRS-1.

### 3.6. QDTS Granules Attenuated the Activation of the MAPK Signal Pathway

**Figure 6:**
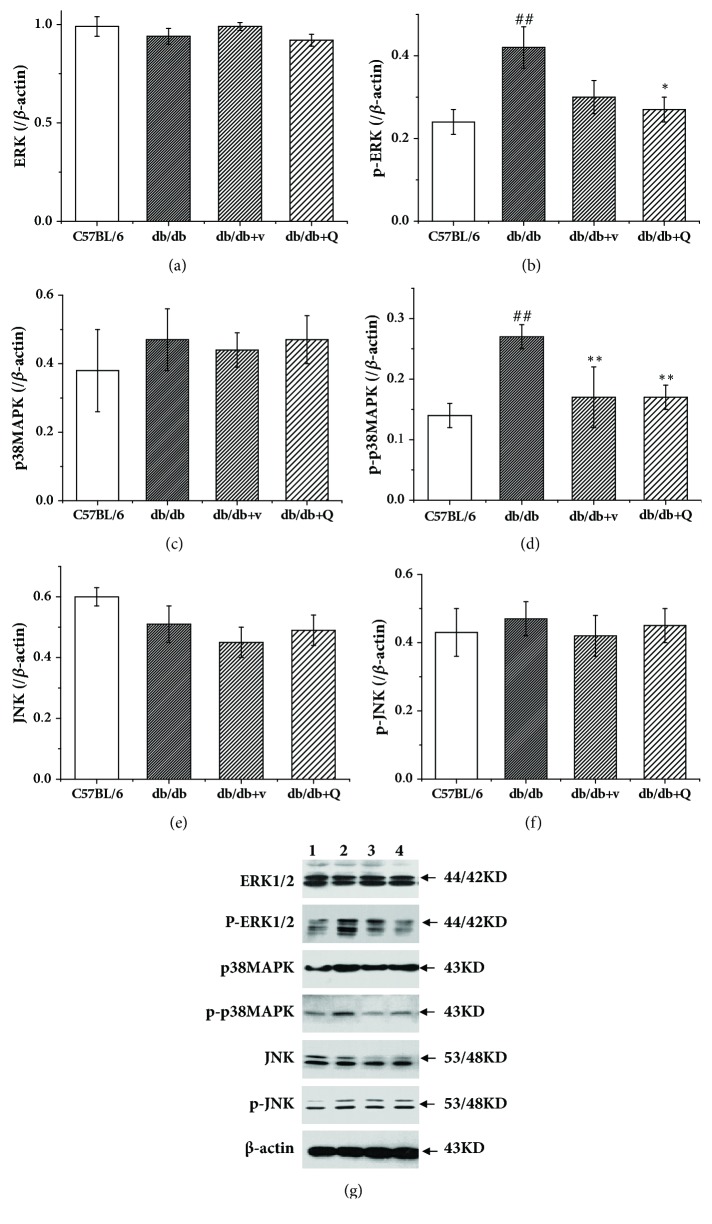
QDTS granules attenuated the activation of the MAPK signaling pathway in the kidney of the db/db mice. (a) Expression of ERK. (b) Expression of phosphorylated ERK. (c) Expression of p38MAPK. (d) Expression of phosphorylated p38MAPK. (e) Expression of JNK. (f) Expression of phosphorylated JNK. (g) Western blot of the expression of EPK, phosphorylated ERK, p38MAPK, phosphorylated p38MAPK, JNK, and phosphorylated JNK. Lane 1, the C57BL/6 group; Lane 2, the db/db model group; Lane 3, the db/db + v group; Lane 4, the db/db + Q group. C57BL/6, the normal control group; db/db, the diabetic nephropathy (DN) model group; db/db + v, the valsartan intervention group; db/db + Q, the QDTS granules intervention group. n = 4 for each group. ^##^*P* < 0.01 compared with the normal control, ^*∗*^*P* < 0.05 compared with the DN model group, and ^*∗∗*^*P* < 0.01 compared with the DN model group.

ERK, p38MAPK, and JNK are the major members of the mitogen-activated protein kinase (MAPK) family. It has been shown that MAPK family members can promote the phosphorylation of the serine residues of IRS-1 and as a result inhibit the phosphorylation of the tyrosine residues of IRS-1 [10, 11]. The present study detected the protein expression of ERK, p38MAPK, JNK, and the respective phosphorylated forms in renal tissues of the C57BL/6 and db/db mice by western blot. Compared with the C57BL/6 mice, there were no significant changes in protein expression of ERK, p38MAPK, and JNK in the db/db mice (Figures 6(a), 6(c), and 6(e)). However, the phosphorylation level of ERK and p38MAPK was significantly elevated (Figures 6(b) and 6(d)). The results indicated that the activation of ERK and p38MAPK was enhanced in the db/db mice showing disease manifestations of DN, and this enhancement effect of DN can be reversed by QDTS granules or valsartan. However, there was no difference in the phosphorylation level of JNK between the C57BL/6 and the db/db mice, and the intervention of QDTS granules or valsartan had no effect on the activation process of JNK (Figure 6(f)).

## 4. Discussion

The db/db mouse is an animal model for spontaneous type 2 DM derived from a single autosomal recessive mutation of the leptin receptor gene. Thus, the db/db mice show the characteristic obesity and enormous appetite; hyperinsulinemia, insulin resistance, and hyperglycemia occur at the age of 2-4 weeks; the secretory function of *β*-cells gradually declines around the age of 4-6 months and the mice exhibit an absolute deficiency in insulin [25]. This process is very similar to the progression of human type 2 diabetes. From the age of 12-14 weeks, the db/db mice can also show manifestations of early stage renal injury, such as glomerular capillary loop hypertrophy, increased mesangial matrix, and glomerular basement membrane (GBM) thickening, as well as the increasing of UAE, but renal failure rarely occurs [26]. In this study, the body weight and blood glucose of the db/db mice were higher than that of the C57BL/6 mice, and the insulin resistance index increased (Figure 1). The UAE in the db/db mice had already increased before intervention and continued to rise over the following 12 weeks. However, there were no significant changes in serum Cr or BUN (Figure 2). After the intervention of valsartan and QDTS granules, the UAE in the db/db mice decreased significantly, but no improvement was observed on body weight and blood glucose level. This suggests that QDTS granules, like valsartan, may reduce diabetic kidney injury through a nonhypoglycemic mechanism.

There are three signaling pathways activated by leptin, including JAK/STAT (Janus kinase/signal transducer and activator of transcription), MAPK, and PI3K (phosphatidylinositol 3-kinase) pathways. As described before, PI3K is also one of the main downstream pathways of insulin signaling. Leptin resistance may cause an increase in insulin requirement and insulin resistance [27]. Studies have shown that the activation of the PI3K/Akt is inhibited in the liver and the muscles of the db/db mice [28, 29]. The results of the present study showed that, compared with the C57BL/6 mice, the activation of PI3K/Akt signaling pathway in the renal tissue of the db/db mice was inhibited (Figure 4), and QDTS granules reduced the insulin resistance index of the db/db mice (Figure 1) and increased the phosphorylation level of PI3K and Akt (Figure 4). The evidence suggested that the decreased activation of the PI3K/Akt signaling pathway has a negative effect on the development of DN. Mice with specific knockout of the podocyte insulin receptor gene could have proteinuria, as well as typical pathological manifestations of DN, while the blood glucose remained normal [7]. Further, the fact that thiazolidinediones (TZDs), a class of insulin sensitizer, can reduce proteinuria of patients with DN by a nonhypoglycemic mechanism has provided another piece of convincing evidence [30]. Therefore, the kidney injury of DM reduced by QDTS granules is presumably via promoting the activation of the PI3K/Akt signaling pathway.

The MAPK signaling pathway mainly consists of the extracellular-signal regulated protein kinase (ERK), p38MAPK, and c-Jun amino-terminal kinase (JNK) pathways. It has been suggested that there may be some connections between the MAPK and the PI3K/Akt signaling pathways. Various studies indicated that the three MAPK signaling pathways could inhibit the transduction of insulin signaling by different mechanisms [31, 32]. It has been found that fibroblast growth factor-1 (FGF-1) elevated the phosphorylation level of the MAPK pathway in hippocampal neurons and at the same time downregulated the phosphorylation level of Akt. In addition, when phosphorylation of the MAPK pathway reached the maximum level, the phosphorylation level of Akt was at its lowest, suggesting an interaction between MAPK kinase (MEK-1/2) and PI3K [33, 34]. It is inferred that there is a certain balance between the activation of the MAPK and PI3K signaling pathways. This is very similar to the concept of the “yin-yang balance" in the theory of TCM. In the current study, we tried to explore whether the mechanism of the therapeutic effect of QDTS granules on the kidney injury of DN is the balance between the PI3K and MAPK signaling pathways. The results obtained in the present study revealed that the unbalance state of MAPK/PI3K phosphorylation could be reversed by QDTS granules, suggesting that QDTS granules may reduce the DN renal injury by downregulating the activation of MAPK, upregulating the activation of PI3K, and consequently restoring the balance between the PI3K and MAPK signaling pathways.

Therefore, understanding the link between the MAPK and PI3K signaling pathways becomes important. It is known that autophosphorylation of the insulin receptor tyrosine kinase occurs after combining with insulin and then activates IRS-1. Subsequently the tyrosine (Tyr) residues of IRS-1 are phosphorylated and serve to recruit proteins containing the Src homology 2 domain, including class I-PI3K molecules; hence, the phosphorylation of IRS-1 on the tyrosine residues is the important prerequisite for the activation of downstream signaling pathways. In type 2 DM, the excessive phosphorylation of serine (Ser) residues of IRS-1 inhibits the phosphorylation of the Tyr residues [34, 35]. It has also been reported that over activated MAPK could increase the phosphorylation of the Ser residues of IRS-1 [10, 11].

However, not all of the Ser residues of IRS-1 have an inhibitory effect on insulin signaling. Some studies have suggested that certain Ser residues can inhibit Tyr phosphorylation of IRS-1 due to their proximity to phosphotyrosine-binding domains (PTB) (e.g., murine Ser302, Ser307; human Ser307, Ser312) or PI3K binding site (e.g., murine Ser612; human Ser616). It has been found that when Ser302, Ser307, and Ser612 of IRS-1 were changed to alanine (Ala) by point mutation in mice at the same time, phosphorylation at these three sites was blocked; the transgenic mice showed an improved glucose tolerance and insulin sensitivity, which was accompanied by elevated phosphorylation levels of the Tyr residue of IRS-1 and PI3K [36]. The present study revealed that compared with the C57BL/6 mice, there was no significant change in the expression of IRS-1 protein and the phosphorylation of Ser307 in the db/db mice, but the phosphorylated Tyr896 of IRS-1 decreased, and the ratio of Ser307/Tyr896 increased consequently (Figure 5). These results indicated that the phosphorylation balance of certain Tyr and Ser residues of IRS-1 is disturbed in type 2 DM. However, for Ser302 of IRS-1, some studies have drawn different conclusions. For example, it has been suggested that the phosphorylation of Ser302 of IRS-1 plays a positive role in normal insulin signaling [8, 37].Nevertheless, in the present study, the expression of phosphorylated Ser302 of IRS-1 in the db/db mice decreased, but the ratio of Ser302/Tyr896 showed no significant change. Therefore, the phosphorylation of the Ser residues of IRS-1 is regulated by a variety of complex factors. The final determinant of the activity of insulin signaling in tissues is not the phosphorylation level of these Ser residues, but the phosphorylation balance between the Ser and the Tyr residues. Therefore, we hypothesized that QDTS granules reduced the kidney injury by decreasing the ratio of phosphorylated Ser307/Tyr896 of IRS-1. In addition, valsartan could also restore the phosphorylation balance between Ser307 and Tyr896 by increasing the phosphorylation level of Tyr896 rather than decreasing the phosphorylation level of Ser307, which is consistent with a previous study [38].

## 5. Conclusions

In conclusion, the present study evaluated the therapeutic effect of QDTS granules on DM kidney injury and insulin resistance, revealing that QDTS granules could restore the phosphorylation balance between the Ser and Tyr residues of IRS-1 via inhibiting the activation of MAPK pathway. The current views hold that the positive or negative feedback mechanism or the concept of a regulatory network in molecular biology is very similar to the concept of “yin and yang" used in the theory of TCM [12]. Therefore, the therapeutic effect of QDTS granules is a good embodiment of the TCM idea of “searching for the primary cause of disease in treatment" and “regulating the balance between yin and yang”.

## Data Availability

The data sets used and analyzed during the current study are available from the corresponding author on reasonable request.
